# Validation of the clinical frailty score (CFS) in French language

**DOI:** 10.1186/s12877-019-1315-8

**Published:** 2019-11-21

**Authors:** Paul Abraham, Delphine S. Courvoisier, Cedric Annweiler, Cliff Lenoir, Thomas Millien, Francoise Dalmaz, Hans Flaatten, Rui Moreno, Steffen Christensen, Dylan W. de Lange, Bertrand Guidet, Karim Bendjelid, Bernhard Walder, Bernardo Bollen Pinto

**Affiliations:** 1 Department of Acute Care, Geneva University Hospitals, Geneva, Switzerland; 2 Faculty of Medicine and Science, Claude Bernard University, Lyon 1, Villeurbanne, France; 3 Geneva Hemodynamic Research Group, University of Geneva, Geneva, Switzerland; 4 Quality of care unit, Geneva University Hospitals, Geneva, Switzerland; 5 Department of Geriatric Medicine, Angers University Hospital; Angers University Memory Clinic; Research Center on Autonomy and Longevity; UPRES EA 4638, University of Angers, Angers, France; 6 Department of Medical Biophysics, Robarts Research Institute, Schulich School of Medecine and Dentistry, the University of Western Ontario, London, ON Canada; 7 Department of Anaesthesia and Intensive Care, Haukeland University Hospital, Bergen, Norway; 8 Unidade de Cuidados Intensivos Neurocríticos, Hospital de São José, Centro Hospitalar de Lisboa Central, Faculdade de Ciência Médicas de Lisboa, Nova Médical School, Lisbon, Portugal; 9 Department of Anaesthesia and Intensive Care Medicine, Aarhus University Hospital, Aarhus, Denmark; 10 Department of Intensive Care Medicine, University Medical Center, University Utrecht, Utrecht, The Netherlands; 11 Assistance Publique-Hôpitaux de Paris, Hôpital Saint-Antoine, service de réanimation médicale, Paris, France; 12 Sorbonne Universités, UPMC Univ Paris 06, Institut Pierre Louis d’Epidémiologie et de Santé Publique, Paris, France; 13 INSERM, Institut Pierre Louis d’Epidémiologie et de Santé Publique, Paris, France; 14 Geneva Perioperative Basic, Translational and Clinical Research Group, Division of Anaesthesiology, Geneva University Hospitals, Geneva, Switzerland

**Keywords:** Older people, Frailty, ICU, Mortality, Severity of illness, Back-translation

## Abstract

**Background:**

Very old critical ill patients are a rapid expanding group. To better understand the magnitude of the challenges involved in intensive care practice for an ageing population and discuss a rational allocation of resources, healthcare practitioners need a reliable evaluation of frailty. In order to promote the adequate use of the Clinical Frailty Scale (CFS) in a wider panel of countries, we aimed to develop, validate and characterise a French (FR) version from the original English (EN) CFS.

**Methods:**

We included participants recruited prospectively for the observational “The very old intensive care patient: A multinational prospective observation study” (VIP Study) at Geneva University Hospitals (FR speaking hospital). A FR version of the CFS was obtained by translation (EN- > FR) and back translation (FR- > EN). The final CFS-FR was then evaluated twice on the same participants with at least a 2-week interval by FR-speaking doctors and nurses.

**Results:**

Inter-rater reliability was 0.87 (95%CI: 0.76–0.93) between doctors for the original CFS version and 0.76 (95%CI: 0.57–0.87) between nurses for the FR version. Inter-rater variability between doctor and nurse was 0.75 (95%CI: 0.56–0.87) for the original version, and 0.73 (95%CI: 0.52–0.85) for the FR version.

Test-retest (stability) with the original vs the FR version was 0.86 (95%CI: 0.72–0.93) for doctors and 0.87 (95%CI: 0.76–0.93) for nurses.

Differences between the evaluations of the CFS-EN and CSF-FR were not different from 0, with a mean difference of 0.06 (95%CI -0.24, 0.36) for the EN version and − 0.03 (95%CI -0.47, 0.41) for the FR version. Average original version ratings were slightly lower than FR version ratings, though this difference did not reach significance: -0.29 (95%CI -0.54, 0.04).

**Conclusion:**

In this prospective cohort of very old intensive care participants we developed and tested the basic psychometric properties (internal consistency, reproducibility) of a French version of the CFS. This manuscript provides clinically meaningful psychometric properties that have not been previously reported in any other language, including in the original EN version.

The French cultural adaptation of this CFS has adequate psychometric properties for doctors or nurses to evaluate frailty in very old intensive care patients.

## Introduction

As Europeans continue to experience increasing lifespans, surgical and perioperative care for the old (> 65) and very old (> 80 years) patients has become commonplace, and is expected to continue to increase in volume and complexity in future decades. Advanced age, as a risk factor in surgery, is the complex combination of an increased probability of comorbidities and “frailty”. Frailty is an insufficiently understood decline in physiological reserve and resilience that may be related to energy production, energy utilization and defective repair mechanisms [1]. Frailty is strongly associated with increased mortality after intensive care (ICU) admission, even when controlling for chronological age and other risk factors [2].

**Fig. 1:**
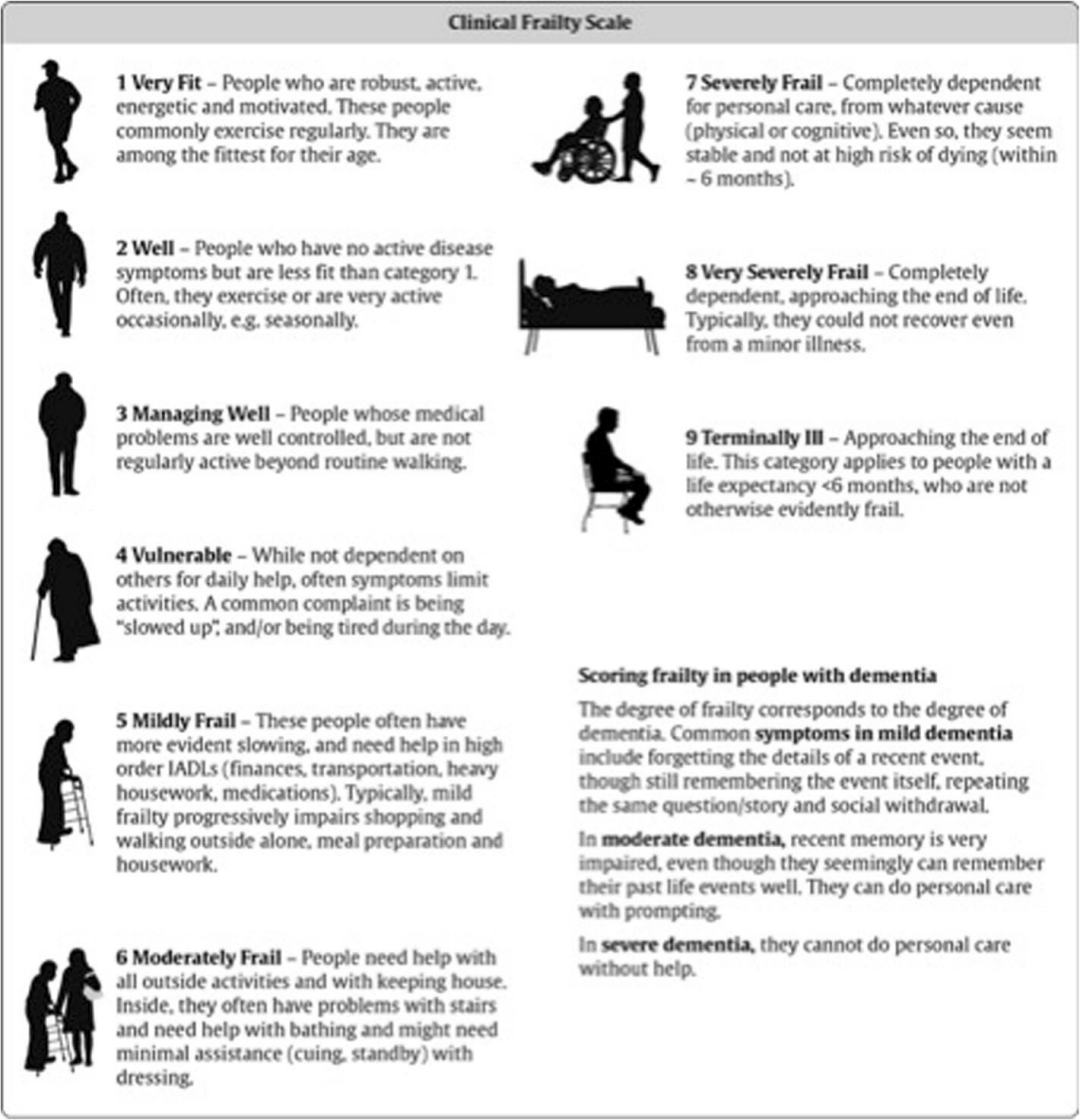
Clinical Frailty Scale, Original EN Version (CFS-EN-original). Permission to use the CSF was granted from Dalhousie University, Ca. May 15. 2017

To better understand the magnitude of the challenges involved in intensive care practice for an ageing population and discuss a rational allocation of resources, healthcare practitioners need a reliable evaluation of frailty [3]. There are multiple instruments to evaluate frailty with a diverse range of complexity, from the 70 items Frailty Index (FI) [4] to the more feasible clinical frailty scale (CFS) [5]. The latter, an ordinal 9-point visual scale in which the assessor makes decisions about the degree of frailty from clinical data, is well correlated with the FI (*r* = 0.80), but much easier to conduct [5]. The score ranges from very fit (CFS = 1) to very severely frail (CFS = 8) and terminally ill (CFS = 9) (Fig. 1). Frailty is usually defined as CFS > 4 [6].

Frailty assessment using tools such as the CFS should be part of the standard multimodal evaluation routinely performed in older adults [5]. However, after a literature search we were only able to identify the original English (EN) version of the CFS validation, thereby limiting its use by clinicians from other native languages. The use of the EN version or a non-validated translation of the CFS by healthcare personnel can result in different assessments and contribute to biases. Items could be answered differently because of differences in translation or culture instead of differences in actual patients’ status, which can lead to inadequate scoring of frailty. Therefore, in order to promote the adequate use of this scale in a wider panel of countries, we aimed to develop, validate and characterise a French (FR) version of the CFS.

## Methods

We included participants recruited prospectively for the observational “The very old intensive care patient: A multinational prospective observation study” (VIP Study) [3] in the Intensive Care and Peri-Interventional Intermediate Care Units at Geneva University Hospitals (FR speaking hospital), between January and July 2017. The study was approved by the Geneva Regional Ethics Committee (Commission cantonale d’éthique de la recherche de Genève, CCER: 2016–01773, President: Professor Bernard Hirschel) that waived the need for informed consent. Observational data were collected according to international ethics standards conforming to the Declaration of Helsinki [7].

### Obtaining a French version for testing

The translation from EN to FR was made in 4 steps by 4 clinicians (2 doctors and 2 nurses) with C2 (Europass) level of both languages, whose native language is FR. The text was then back-translated into EN by 2 independent clinicians (doctor and nurse) with the same language skills whose native language was EN. They were blinded to the original EN version. All translators were aware of the study design.

The original EN (CFS-EN) and EN back-translated versions were then compared qualitatively. Differences or incoherence between the two versions (CFS-EN original and EN-back-translated) were resolved by agreement in order to improve the French translated version.

The FR version was then further assessed by 5 Healthcare workers whose native language is French (nurses and doctors) working in the Geneva intensive or intermediate care units. Their feedback was used to further modify the scale and obtain the definite FR translated version (CFS-FR).

### Characterizing and validating the FR-final version

The CFS was evaluated twice on the same participants with at least a 2-week interval. Evaluators were either of the same profession (nurse or physician) or of differing profession, to assess interjudge agreement within and between professions. The CFS was also assessed twice by the same evaluators, to evaluate test-retest reliability. Furthermore, the scale used was either in the same language or of differing language, to assess whether the ratings were similar with the French, compared to the English version of the scale. Doctors evaluated the English version twice and nurses evaluated the French version twice. Evaluators were blinded to each other’s evaluation.

Criterion validity was assessed by examining the relation of CFS-EN and CFS-FR with mortality at 30-days after ICU admission, using Wilcoxon rank sum test.

Interjudge reliability and test-retest reliability were assessed using intraclass correlation (ICC) and Bland and Altman plot. ICC inter-rater agreement measures were considered poor - Less than 0.40, fair - Between 0.40 and 0.59, good - Between 0.60 and 0.74, excellent - Between 0.75 and 1.00 [8].

## Results

Of the 40 participants recruited to the VIP1 study, the CFS evaluation was performed in 34 participants. In 6 (15%) participants, one or more operators were not able to provide a score due to insufficient data on participant health status prior to ICU admission. These 6 participants were excluded from further analysis. Mortality follow up was completed for all participants. Participants were mostly female (57%) and were on average 84.1 years old.

**Fig. 2:**
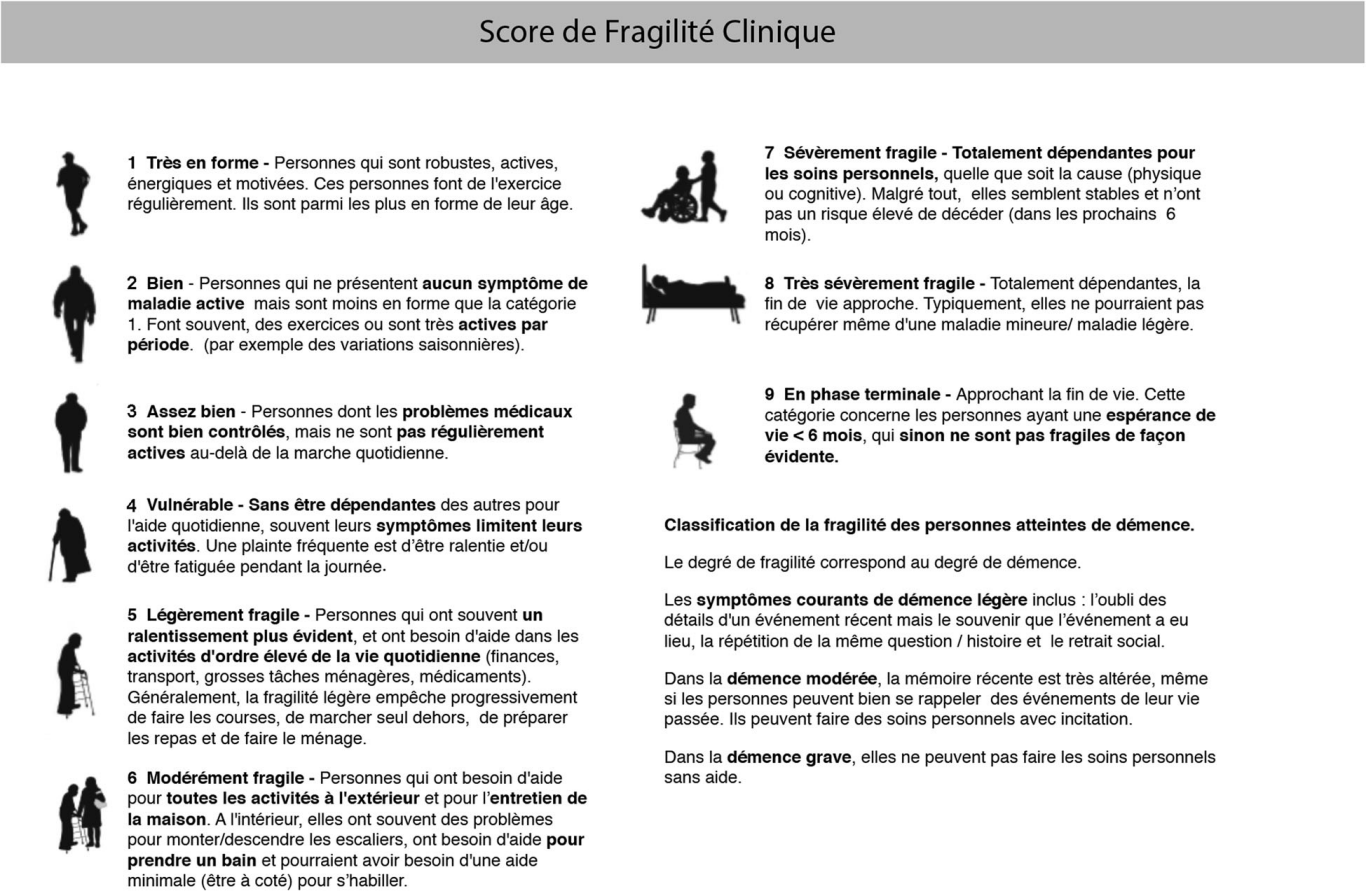
Clinical Frailty Scale, French translated final version (CFS-FR). Permission to use the CSF was granted from Dalhousie University, Ca. May 15. 2017

Inter-rater reliability was 0.87 (95%CI: 0.76–0.93) between doctors for the EN version (Fig. 1), and 0.76 (95%CI: 0.57-0.87) between nurses for the FR version (Fig. 2). Inter-rater variability between doctor and nurse was 0.75 (95%CI: 0.56-0.87) for the EN version, and 0.73 (95%CI: 0.52-0.85) for the FR version.

Test-retest (stability) with the EN vs the FR version was 0.86 (95%CI: 0.72–0.93) for doctors and 0.87 (95%CI: 0.76–0.93) for nurses.

**Fig. 3:**
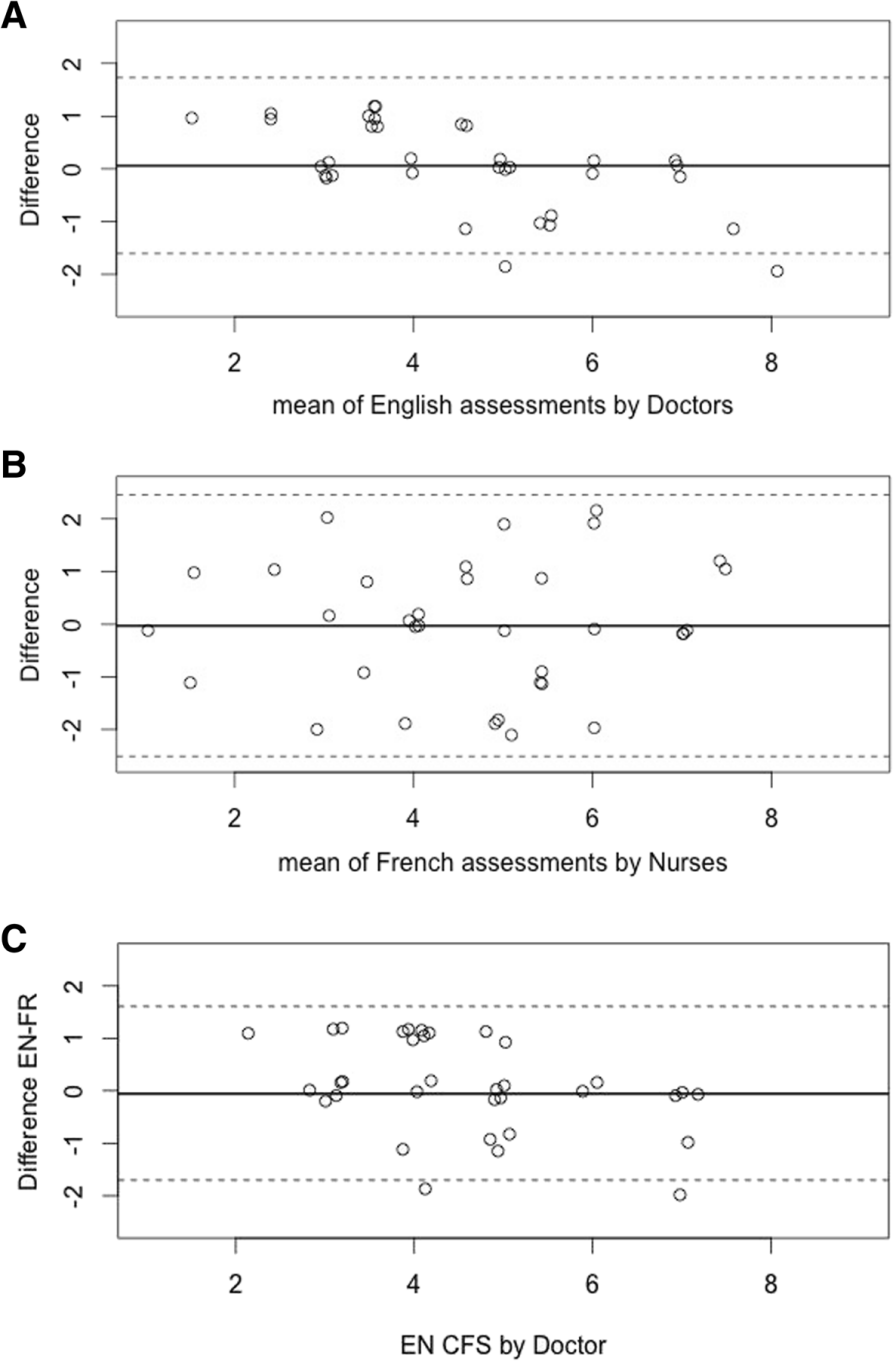
Bland et Altman plot for CFS scoring between 2 independent Doctors with CFS-EN (**a**), between 2 independent Nurses with CFS-FR (**b**), with the EN then FR version by Doctor (**c**)

Differences between the evaluations of the CFS-EN and CSF-FR were not different from 0, with a mean difference of 0.06 (95%CI -0.24-0.36) for the EN version and -0.03 (95%CI -0.47-0.41) for the FR version (Fig. 3a, b). Agreement between the FR and the EN version for doctors was similar (Fig. 3c). Average English version ratings were slightly lower than French version ratings, though this difference did not reach significance: -0.29 (95%CI -0.54-0.04).

There were 15 deaths within 30-days of ICU admission. There were no significant differences in the CFS scores between participants who died within 30 days and participants who survived for either the EN (median survived: 4.7, median died: 4.0, *p* = 0.52) or FR (median survived: 4.7, median died: 4.5, *p* = 0.56) versions.

## Discussion

Overall, the EN and FR versions of the CFS exhibited good to excellent interjudge reliability, between doctors, between nurses, and to a lesser extent between nurses and doctors [8]. The test retest of either the FR or the EN versions showed a good stability. Bland and Altman representation showed a good agreement between doctors (see Fig. 3a).

Only 2 measures differed by more than 2 points with the CFS-EN scale performed by 2 independent doctors. Agreement between nurses with the FR version was fair (see Fig. 3b). Moreover, agreement between the FR and the EN versions for Doctors seemed strong enough to validate this EN-to-FR translation in clinical practice (see Fig. 3c).

As expected, the CFS scores were slightly higher in participants who died than in those who survived, though significance could not be achieved in this small cohort.

This study has some limitations. This is a report of a simple study using a standard forward-back translation method to develop and test a French version of an English questionnaire. The characterization and validation the FR-final version was performed in a relatively small number of participants, as this was a convenience sample using patients enrolled in the larger VIP1 study in Geneva University Hospitals. However, our sample size of 40 patients would allow us to detect an ICC of 0.75 with a half-confidence interval width of 0.25. In 6 patients one or more operators were not able to provide a score due to insufficient data, thus raising the possibility of selection bias. Importantly, all values from the CFS except 9 are represented in the sample; hence in our opinion it is unlikely that the missing patients have an important influence in the validation study considering the range of analyses performed.

## Conclusion

In this prospective cohort of very old intensive care participants we developed and tested the basic psychometric properties (internal consistency, reproducibility) of a French version of the CFS. This manuscript provides clinically meaningful psychometric properties that have not been previously reported in any other language, including in the original EN version [5]. The French cultural adaptation of this CFS has adequate psychometric properties for doctors or nurses to evaluate frailty in very old intensive care patients.

## Data Availability

The datasets used and/or analysed during the current study are available from the corresponding author on reasonable request.

## Abbreviations

CFS: Clinical frailty Scale
CI: Confidence Interval
EN: English
FI: Frailty Index
FR: French
ICC: Intraclass correlation
ICU: Intensive care unit
